# A computational pipeline for the development of multi-marker bio-signature panels and ensemble classifiers

**DOI:** 10.1186/1471-2105-13-326

**Published:** 2012-12-08

**Authors:** Oliver P Günther, Virginia Chen, Gabriela Cohen Freue, Robert F Balshaw, Scott J Tebbutt, Zsuzsanna Hollander, Mandeep Takhar, W Robert McMaster, Bruce M McManus, Paul A Keown, Raymond T Ng

**Affiliations:** 1 NCE CECR Prevention of Organ Failure (PROOF) Centre of Excellence, Vancouver, BC, V6Z 1Y6, Canada; 2 Department of Statistics, University of British Columbia, Vancouver, BC, V6T 1Z2, Canada; 3 Department of Pathology and Laboratory Medicine, University of British Columbia, Vancouver, BC, V6T 2B5, Canada; 4 Immunity and Infection Research Centre, Vancouver, BC, V5Z 3J5, Canada; 5 Immunology Laboratory, Vancouver General Hospital, Vancouver, BC, V5Z 1M9, Canada; 6 Department of Medicine, University of British Columbia, Vancouver, BC, V5Z 1M9, Canada; 7 James Hogg Research Centre, St. Paul’s Hospital, University of British Columbia, Vancouver, BC, V6Z 1Y6, Canada; 8 Department of Computer Science, University of British Columbia, Vancouver, BC, V6T 1Z2, Canada; 9 Department of Medical Genetics, University of British Columbia, Vancouver, BC, V6T 1Z3, Canada; 10 Institute for HEART+LUNG Health, Vancouver, BC, V6Z 1Y6, Canada; 11 Department of Medicine, Division of Respiratory Medicine, University of British Columbia, Vancouver, BC, V5Z 1M9, Canada; 12 Department of Statistics and Actuarial Science, Simon Fraser University, Burnaby, BC, V5A 1S6, Canada

**Keywords:** Biomarkers, Computational, Pipeline, Genomics, Proteomics, Ensemble, Classification

## Abstract

**Background:**

Biomarker panels derived separately from genomic and proteomic data and with a variety of computational methods have demonstrated promising classification performance in various diseases. An open question is how to create effective proteo-genomic panels. The framework of ensemble classifiers has been applied successfully in various analytical domains to combine classifiers so that the performance of the ensemble exceeds the performance of individual classifiers. Using blood-based diagnosis of acute renal allograft rejection as a case study, we address the following question in this paper: *Can acute rejection classification performance be improved by combining individual genomic and proteomic classifiers in an ensemble?*

**Results:**

The first part of the paper presents a computational biomarker development pipeline for genomic and proteomic data. The pipeline begins with data acquisition (e.g., from bio-samples to microarray data), quality control, statistical analysis and mining of the data, and finally various forms of validation. The pipeline ensures that the various classifiers to be combined later in an ensemble are diverse and adequate for clinical use. Five mRNA genomic and five proteomic classifiers were developed independently using single time-point blood samples from 11 acute-rejection and 22 non-rejection renal transplant patients. The second part of the paper examines five ensembles ranging in size from two to 10 individual classifiers. Performance of ensembles is characterized by area under the curve (AUC), sensitivity, and specificity, as derived from the probability of acute rejection for individual classifiers in the ensemble in combination with one of two aggregation methods: (1) Average Probability or (2) Vote Threshold. One ensemble demonstrated superior performance and was able to improve sensitivity and AUC beyond the best values observed for any of the individual classifiers in the ensemble, while staying within the range of observed specificity. The Vote Threshold aggregation method achieved improved sensitivity for all 5 ensembles, but typically at the cost of decreased specificity.

**Conclusion:**

Proteo-genomic biomarker ensemble classifiers show promise in the diagnosis of acute renal allograft rejection and can improve classification performance beyond that of individual genomic or proteomic classifiers alone. Validation of our results in an international multicenter study is currently underway.

## Background

With the advancement of whole-genome technologies and unbiased discovery approaches such as microarrays and mass spectrometry, molecular biomarker panel development has attracted much attention and investment in the past decade. Given that biomarker panels may be valuable for prognosis, diagnosis or prediction of a medical condition, or for efficacy and safety of a treatment option [1-3], many teams have embarked on biomarker panel development projects and programs with the aim of clinical utility and health care benefits.

The mission of the NCE CECR Centre of Excellence for Prevention of Organ Failure (PROOF Centre) is to develop biomarker panels for heart, lung and kidney conditions along the life cycle from risk to presence, progression and variable responses to clinical interventions including pharmacotherapies. Its flagship program is the Biomarker in Transplantation initiative, which began in 2004. One branch of the work focuses on renal allograft rejection, which is harnessed in this paper as an illustrative case study. Samples from this study are of one of two types: acute rejection (AR) or non-rejection (NR), representing binary classification tasks. Acute renal allograft rejection in transplant patients in Canada occurs in approximately 10-20% of transplant patients within the first 12 weeks post-transplant. Acute rejection is a serious problem that leads to kidney failure and graft loss if untreated and recurrent. Early detection of acute rejection with a highly sensitive test followed by appropriate treatment is thus of paramount importance; similarly, the exclusion of acute rejection with a highly specific test followed by tailoring of immunosuppressive therapy will benefit many patients by reducing toxic side-effects. Acute rejection is currently diagnosed by tissue biopsy, an invasive procedure that requires subjective grading by pathologists to determine if and to what degree acute rejection is present in the tissue sample [4]. A promising alternative to tissue biopsy, one which we have pursued since 2004, is the use of blood-based biomarkers for diagnosing acute rejection. We reported the first such genomic biomarker panel in Transplantation [5] and a proteomic panel in Molecular&Cellular Proteomics [6]. With successful replication of our early results, we participated in a Voluntary Exploratory Data Submission to the USA FDA. A multi-national observational trial on refined biomarker panels is now in its late stages, with the goal of obtaining regulatory approval from the USA FDA and Health Canada.

This paper will first present an established computational biomarker development pipeline for genomic and proteomic data. The pipeline begins with data acquisition (e.g., from bio-samples to microarray data), quality control, statistical analysis and mining of the data, and finally various forms of validation. Several groups, including ours, have explored blood-based genomic and proteomic classifiers of acute rejection in kidney and heart transplant recipients with promising results [5-11]. However, the potential of combining genomic- and proteomic-based classifiers in an effective manner remains largely unknown. Second, we describe an ensemble approach for building proteo-genomic biomarker panels. An intuitive strategy for building such panels is to merge genomic and proteomic data and apply a single-platform analysis strategy to the merged data set [12,13]. Unfortunately, with this approach, one encounters challenges related to scaling and normalization, especially with the large differences in the distribution of the data values between the two platforms. In addition, due to differing signal strengths between genomic and proteomic data, it is likely for data from one platform to dominate the final classifier panel, masking what might be a potentially valuable contribution from the second data type. Although these issues have been addressed by potential solutions, such as the promising approach taken by mixOmics tools that incorporate partial least squares and canonical correlation analysis [14], a different path is described in this paper. Fully developed individual classifiers are combined in an ensemble [15-17], thus avoiding the aforementioned issues while allowing for an intuitive interpretation and straight-forward implementation.

## Methods

### Biomarker development pipeline

**Figure 1:**
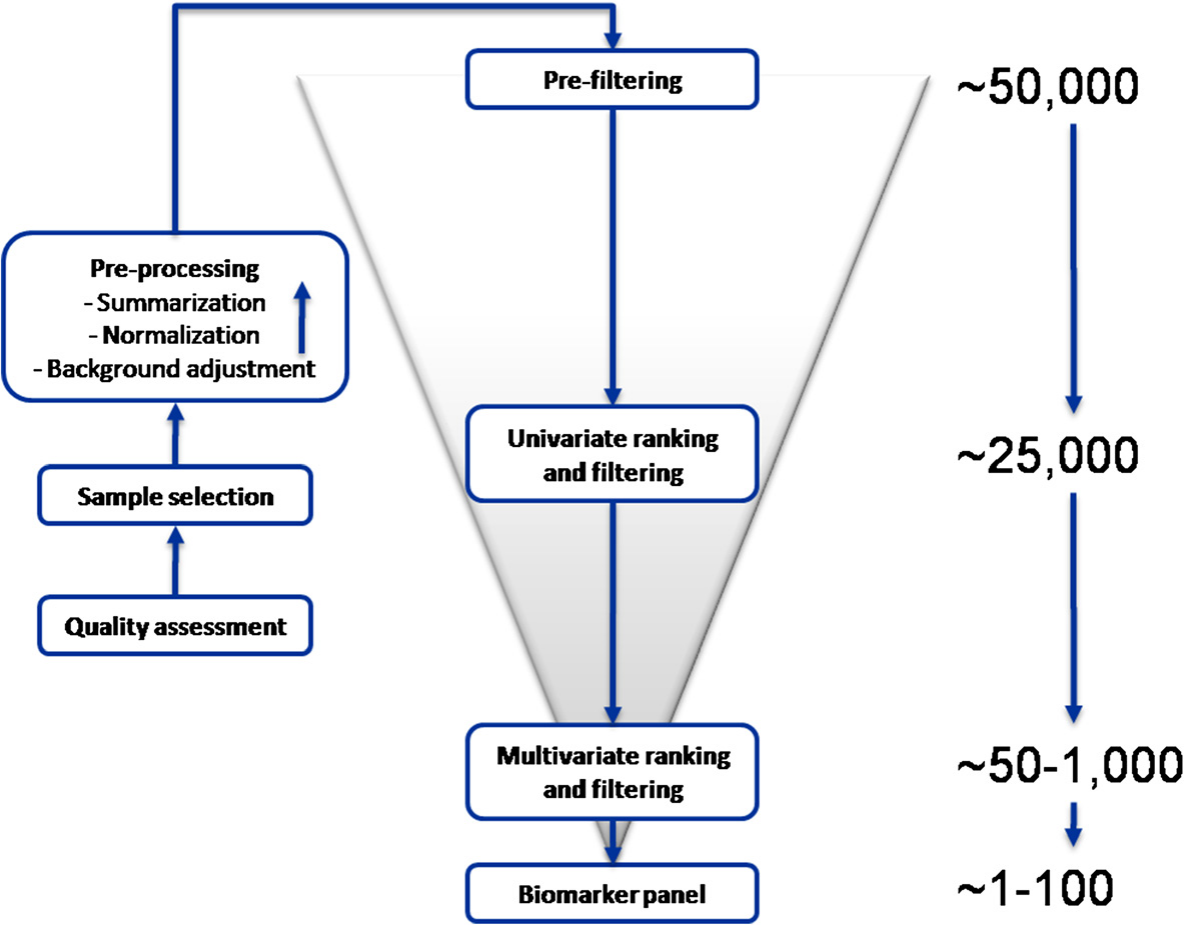
**Schematic representation of the biomarker development pipeline for genomic microarray data.** The analysis starts with a pre-filtering step applied to the full pre-processed data set (54613 probe sets from the Affymetrix Human Genome U133 Plus 2 GeneChip) on top of the funnel, followed by uni- and multivariate ranking and filtering steps before arriving at a biomarker panel. The numbers on the right indicate the number of features (probe sets) at each step. The biomarker development pipeline for proteomic data looks similar except that data sets are typically smaller and proteomic-specific pre-processing steps need to be applied.

The biomarker development process represents a series of sequential steps that can be described as a computational pipeline. Figure 1 shows the genomic biomarker development pipeline, with initial data quality assessment, sample selection, and pre-processing steps on the left, and main analysis components such as pre-filtering, uni- and multivariate ranking and filtering steps in the center. The numbers on the right represent the number of features (e.g., probe sets in the genomic case) that correspond to each analysis step. The purpose of pre-filtering, uni- and multivariate ranking, and filtering steps is to reduce the number of features to be used in the classification model, while selecting relevant features for the classification task. This final list of features represents the biomarker panel which typically ranges in size from 1–100 features.

The analysis of proteomic data requires some proteomic-specific analytical steps that are beyond the scope of this article, including data assembled from untargeted lists of identified protein groups, imputation of missing values, and quality assessment of protein identification parameters [18]. Regardless, the main aims of the analyses undertaken at the different steps of the proteomics and the genomics pipeline are essentially the same. Briefly, at the discovery stage, the proteomics computational pipeline utilizes a combination of appropriate univariate and multivariate statistical methodologies to identify a panel of candidate biomarker proteins. The quality of the identified list of markers is evaluated looking at protein identification parameters and examining the existence of potential confounding factors. In previous studies based on iTRAQ-MALDI-TOF/TOF technology, the total number of identified protein groups was about 1500. However, due to undersampling commonly seen in shotgun proteomics studies, only about 10% of these protein groups were consistently detected in all the samples involved in a particular analysis. Thus, the proteomic analysis data sets from this technology were smaller than the genomic one described in Figure 1.

#### Quality assessment

It is important to detect quality issues to prevent them from entering the biomarker development pipeline and negatively affecting analysis results. The quality of samples is therefore assessed as the first step. Only samples that did not raise quality concerns are included in the analysis, otherwise samples are reanalyzed using a different aliquot of the same sample. For Affymetrix Human Genome U133 Plus 2 GeneChip microarray experiments, quality assessment is through visual inspection of RLE, NUSE and weight plots produced with the AffyPLM-package. Other options include the MDQC-package (developed at the PROOF Centre) and the arrayQualityMetrics-package in R [19,20]. Quality control of the plasma depletion step and the acquired iTRAQ data have been previously described [6], which examines the reproducibility of sample handling procedures, the confidence on the identified protein identities to be analyzed as well as their levels.

#### Sample selection

Analysis samples are selected by a domain expert working with a statistician to ensure that a statistically sound analysis can be performed on samples that are relevant to the study question. Group sizes are reviewed to ensure a reasonable design in regards to balance, possible confounders (typical examples include gender, age, ethnicity), and power of the study. The domain expert is responsible for choosing samples that represent the conditions of interest. For the two-group acute kidney rejection case study that is used as an example throughout this paper, a nephrologist confirmed the rejection status of individuals with acute rejection (AR) based on biopsy information, and selected control cases with clinical and demographic characteristics similar to those of rejection cases. The time of blood collection relative to start of rejection treatment in AR patients is an important factor [21], and was taken into account during sample selection. The presented case study is based on a prospective longitudinal design, which required a sample selection step as described in Figure 1. Depending on the specific experimental design, a sample selection step might not be needed in general.

#### Pre-processing

Depending on the type of data, specific pre-processing steps are applied to prepare the raw data for subsequent statistical analysis. In the case of Affymetrix microarray experiments, raw data represents expression values for probes on the array. These values are provided in CEL-files together with other information about the experiment. Pre-processing in this case includes background adjustment, normalization and summarization of probe information into probe sets that can be mapped to genes. This process transforms raw CEL-files into a data matrix of probe set values for all analysis samples. We have used the Robust Multi-Array Average (RMA)-pr1ocedure in Bioconductor as implemented in the RMA- and RefPlus-packages to perform these steps but other methods can be substituted, for example GCRMA or Factor Analysis for Robust Microarray Summarization (FARMS) [22-25]. The normalization can use an expanded sample set to provide increased performance and stability of the pre-processing procedures, e.g., by including all available microarray samples at different time points for the selected patients in the RMA-normalization procedure.

#### Prefiltering

Not all features in a data set carry useful information. Probe sets with little variation and low expression for example are dominated by noise that can negatively affect the statistical analysis, depending on the methods used. The main goal of the pre-filtering step is therefore to remove features with little variation across analysis samples, independent of sample class, before applying univariate ranking and filtering methods on the remaining features. For that purpose a quantile-based filter was applied to the kidney rejection case study which ranked all samples according to an empirical central mass range (ECMR) as given in Eq.(1) where *f*_1_ is the fraction of the smallest class, e.g. $f_{1}=\min\left(\frac{N_{\mathrm{AR}}}{N_{\mathrm{AR}}+N_{\mathrm{NR}}},\frac{N_{\mathrm{NR}}}{N_{\mathrm{AR}}+N_{\mathrm{NR}}}\right)$ in the 2-class classification problem of acute renal allograft rejection, and then removed all features with values below the median ECMR.

(1) $$\begin{array}{c} \mathrm{ECMR}\left(x\right)=\mathrm{quantile}\left(x,\;1-\frac{f_{1}}{2}\right)\\ \;-\mathrm{quantile}\left(x,\frac{f_{1}}{2}\right) \end{array}$$

For the genomic data from the Affymetrix Human Genome U133 Plus 2 GeneChip, this approach removes half of the 54,613 probe sets. If a more stringent pre-filter is desired, one could for example remove 75% of features with the lowest ECMR. The inter-quartile range (IQR) is a special case of the ECMR with *f*_1_=0.5, i.e., IQR and ECMR are the same for balanced class sizes. For unbalanced class sizes the ECMR-based filter allows variation in the smaller class to enter the calculation of the quantile range. Other pre-filtering options include application of an absolute count cut-off that requires at least *k* samples to have an expression above a fixed threshold, which would address concerns regarding the impact of dependencies between pre- and univariate filters and the ability to control type-I error rates [26]. The choice of threshold in any of these methods represents a trade-off between allowing more potential biomarkers to pass the filter and at the same time adding more noisy features, which increase the chance of identifying false biomarkers down-stream.

#### Univariate ranking and filtering

Having a large number of features in a biomarker panel is typically not practical, as diagnostic or predictive tests in clinical applications are commonly based on a small number of relevant markers. In fact, many currently applied laboratory tests are based on single markers. In addition, some classification models pose statistical constraints on the number of features that they can incorporate, e.g., a Linear Discriminant Analysis (LDA) classification model has to be based on fewer features than the number of training samples. For that reason a univariate ranking and filtering step is applied to reduce the number of candidate features to be included in the classification model.

The univariate ranking step calculates a measure of class differentiation ability for each individual feature that passed the pre-filtering stage. Moderated *t*-tests are commonly used for determining differentially expressed features when sample sizes are small. Examples are the limma-package in Bioconductor or the Signficance Anaysis of Microarrays (SAM) tool [27,28]. These tests return adjusted p-values or false discovery rates (FDR) that account for multiple hypothesis testing by applying permutation tests (SAM), Bonferroni, Benjamini and Hochberg, or other methods, which is generally recommended for –omics data [29,30]. The limma package includes an empirical Bayes method that moderates the standard errors of the estimated log-fold changes. This approach results in more stable inference and improved power, especially for experiments with small numbers of arrays [27].

Various combinations of FDR cut-offs and fold-change thresholds are applied to produce reduced lists of candidate features that serve as input for the subsequent multivariate ranking, filtering and supervised learning steps. In addition, lower and upper counts for the number of features are sometimes imposed to ensure a minimum and/or maximum number of features in the returned list.

#### Multivariate ranking and filtering

It might be desirable in some instances to filter a list of features that are relevant as a group without requiring all of them to be relevant individually. Multivariate ranking is achieved by applying a multivariate scoring method that orders features by method-specific weights. Examples are support vector machines (SVM) where squared weights from the SVM model are used, or Random Forest (RF) which provides a feature-importance measure. The multivariate filtering step simply applies a cut-off regarding the number of ranked features to include.

The steps described above are put together in the order shown in Figure 1 to develop a biomarker panel. The final product in terms of class prediction, e.g. acute rejection versus non-rejection, is a classification model based on a biomarker panel in combination with a supervised learner. The requirements for a supervised learner are that it has to be able to (1) train its classification model based on a training set representing pairs of features (input) and response (output), and (2) return a class probability or score for the different response types given a test case, i.e., a set of input features. Not all steps in the center portion of Figure 1 are performed every time. For example, the multivariate ranking and filtering step may be skipped, and the output from the univariate steps is then used to directly define the biomarker panel. It is possible that a classification model applies an additional feature selection step, e.g., Elastic Net [31].

For the binary classification task of separating acute rejection from non-rejection samples, four supervised learning methods were applied: Support Vector Machine (SVM) with linear kernel, Linear Discriminant Analysis (LDA), Elastic Net (EN), and Random Forest (RF) [31-34]. Where applicable, algorithm parameters were tuned for model selection. Additional methods such as PAM (Shrunken Centroids) [35] or sPLS-DA (mixOmics) [14] have been explored for other data sets at the PROOF Centre.

### Model assessment and selection

Performance of classification models needs to be estimated for model assessment and selection. For this purpose, it is common practice to split a data set into 3 parts: (1) training, (2) validation and (3) testing, with suggested splits being 50%, 25% and 25%, respectively [34]. A set of candidate models is first trained on the training set. One of the candidate models is then selected by comparing performances on the validation data, and the performance of the selected model is finally assessed on the test data. In many cases however, particularly in the high-throughput genomic and proteomic arena, data sets suffer from low sample size and cross-validation or boot-strap methods are typically applied to avoid excluding too many samples from the training set.

For the present case study, nested leave-one-out cross-validation (LOO-CV) was used in combination with minimum misclassification error for model selection and assessment. The outer loop was used for model selection while the nested loops were used for model assessment by averaging performance over *k* models that were tuned in the inner loops of the nested cross-validation procedure. Model parameters were tuned for Elastic Net (*lambda*) and LDA (number of features), while the default *cost* parameter in SVM and default settings for *mtry* and *node-size* parameters were used in Random Forest, since these parameters had little impact on the classification performance in the given data sets. In general, it is advisable to tune these parameters and study their effects on classification performance to decide whether tuning is necessary. Estimators based on LOO-CV are known to have low bias but large variance. An alternative to nested LOO-CV, especially for larger sample sizes, is based on averaging performances over multiple *k*-fold CV partitions.

In general, models with multiple parameters require multi-parameter optimization. This is not straightforward especially when sample sizes are small and different areas of the multi-parameter plane show the same or similar performances. In these cases it is not clear which parameter combination should be chosen. One solution is to fix all but one parameter and select a model based on tuning that parameter. For example, Elastic Net has two parameters, *alpha* and *lambda*, where *alpha* is typically fixed to select a trade-off between Lasso penalization and ridge regression, while *lambda* is varied to tune the model.

In addition to misclassification error, sensitivity, specificity and area under the ROC curve (AUC) were determined. Misclassification error, sensitivity and specificity depend on the probability cut-off used. For example, if a sample has a predicted probability of 0.4 of being an AR, it would be misclassified using a cut-off of 0.5 but correctly classified using a cut-off of 0.3. Misclassification error is the fraction of misclassified AR and NR samples. All reported misclassification errors, sensitivities and specificities are based on a 0.5 cut-off. The AUC is a quantitative measurement that averages classification performance over all probability cut-offs, and as such does not depend on any particular cut-off value.

### Ensemble classifiers

**Figure 2:**
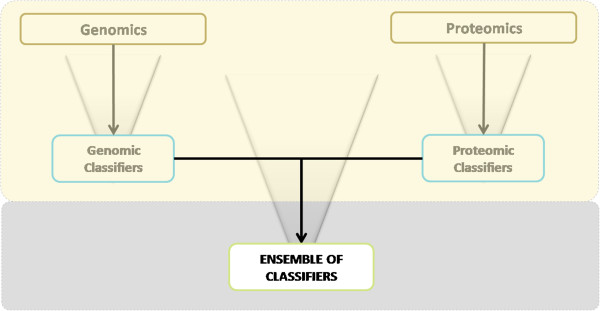
**Schematic overview of ensemble classifiers.** Ensemble classifiers represent a combination of genomic and proteomic classifiers. Individual classifier output is aggregated by either average probability or vote threshold (a modified version of majority vote).

In an effort to integrate multiple classification models, separately developed genomic and proteomic classifiers were combined in an ensemble of classifiers as shown in Figure 2. Ensemble classification methods have been applied in a variety of fields with promising results [15,33,34,36]. Ensembles often combine predictions from a large number of different individual classifiers to produce a final classification that is based on specific aggregation methods, e.g., average vote. The motivating idea behind ensembles is that inclusion of a diverse set of classifiers ensures representation of various aspects of the underlying system, while a single classifier typically has limited focus. For example, a genomic classifier might focus mainly on an immunological signature in whole blood, while a proteomic classifier might focus on an inflammation signature in plasma.

Proteo-genomic ensembles combine classifiers from genomics and proteomics in an effort to improve performance and robustness of predictions. Each ensemble consists of a set of genomic and proteomic classifiers that are characterized by a biomarker panel, i.e., a list of probe sets or protein groups. All classifiers produce a probability of acute rejection (AR) when given an unknown sample. Predicted class probabilities from individual classifiers were aggregated using one of two methods: Average Probability (AP) or Vote Threshold (VT). The AP aggregation method averaged class probability for a specific sample from all individual classifiers in the respective ensemble. Ensemble AUC and other performance measures were then derived from these average probabilities. The VT aggregation method represents a modified majority vote approach that can be applied to binary classification tasks with only two classes G1 and G2. The predicted class from each classifier is interpreted as a vote for that class; if the number of votes for G1 exceeds a fixed threshold, then the predicted class is G1, otherwise it is declared G2.

Ensembling of classifiers is well-studied in the literature [36,37]. In [36], the analysis of ensembling is extended to imbalanced and high-dimensional data (e.g., tens of thousands of probe sets). The analysis indicates that the more "independent" the individual classifiers are, the larger the expected performance gain of the ensemble. This is particularly relevant to integrating molecular signals from whole blood RNA and plasma proteins.

Prior to the case study described in this paper, blood samples were collected from renal allograft recipients in the Biomarkers in Transplantation initiative- [5,6]. Whole-blood RNA samples were analyzed with Affymetrix Human Genome U133 Plus 2.0 arrays (genomic) and plasma samples were analyzed with iTRAQ MALDI-TOF/TOF Mass Spectrometry (proteomics). The two data sources are derived from different compartments of peripheral blood and focus on two separate types of biological material, i.e., leukocyte cellular RNA and plasma proteins. Perhaps not surprisingly, signals detected by genomic analysis are different from those detected by proteomic analysis, although both types of signals are consistent with the current understanding of the pathogenesis of acute rejection injury. In particular, differentially expressed genes represent three major biological processes related to immune signal transduction, cytoskeletal reorganization, and apoptosis [5], and differentially expressed proteins represent biological processes related to inflammation, complement activation, blood coagulation, and wound repair [6]. This diversity in biological signals is maintained in individual genomic- and proteomic-based acute rejection classifiers, and is a desired property in ensemble classifiers. In general, ensemble classifiers demonstrate improved classification performance when individual classifiers in the ensemble represent independent experts [17,38,39].

Although the current case study focuses on combining genomic with proteomic data, the ensemble framework is more general in nature and does not need to be restricted to these types of data. A second analysis was performed to show how gene expression could be combined with miRNA classifiers. This analysis was based on publicly available mRNA- and miRNA- data sets from a cancer study [40]. Using the computational pipeline, classifiers for the diagnosis of tumour- versus normal- samples were developed separately for the mRNA- and miRNA- data sets. A number of ensembles were defined and performances for the AP and VT aggregation methods were estimated.

## Results

Genomic and proteomic classifiers were developed independently with the biomarker development pipeline using 32 samples from the same 32 patients (11AR and 21 NR) collected at the same time point. All samples were used for classifier training, and thus no samples remained for classifier testing. As such, validation and calculation of probability-of-AR was done with 32-fold (leave-one-out) cross-validation wherein 32-models were created for each of the genomic and proteomic classifiers separately with one of the samples left out. The classifer then tested the left-out sample and a probability-of-AR was returned. When classifier development included a model tuning step, nested cross-validation was applied to ensure an unbiased estimate of the probability-of-AR.

The 32 samples were used in previous publications that describe the development of the Genomics 1 and Proteomics 1 classifiers with a simplified pipeline [5,6]^a^. Genomic data represent RNA-based gene expression profiles as measured by Affymetrix HG-U133 Plus 2 GeneChips and were pre-processed with RMA using an enlarged pool of 195 genomic samples that were available at different time-points for the 32 patients, plus an additional 20 samples from healthy volunteers, taken from the same biomarker project as described in [5]. An ECMR-based pre-filter shown in Eq. (1) was applied to the subset of 32 analysis samples and returned 27,306 probe sets for the analysis. Expression values were analyzed on the log-base 2 scale.

Proteomic data represent ratios between depleted plasma samples from transplant patients and healthy pooled controls as measured by iTRAQ-MALDI-TOF/TOF methodology and several post-processing steps, including ProteinPilot™ software v2.0 with the integrated Paragon™ Search and Pro Group™Algorithms, and searching against the International Protein Index (IPI HUMAN v3.39) database. A Protein Group Code Algorithm (PGCA; in-house) was used to link protein groups across different iTRAQ experiments by defining global protein group codes (PGC) from multiple runs [6]. There were a total of 1260 PGCs, each of which was detected in at least one sample. Of those, 147 PGCs passed a 75% minimum detection rule filter across the 32 analysis samples^b^.

The number of features and performance characteristics of five genomic and five proteomic classifiers is summarized in Table 1^c^. Performance of individual classifiers as measured by AUC was typically high, and specificity was higher than sensitivity for all classifiers. In addition to the published genomic classifier [5], four additional genomic classifiers based on SVM, RF and EN classification methods were developed [31-34]. Genomics 2 (SVM) and 3 (RF) classifiers were based on the top 50 FDR-ranked probe sets while Genomics 4 and 5 classifiers were based on probe sets selected by Elastic Net from the probe sets with an FDR<0.05 (with an additional constraint of at least 50 but at most 500 probe sets).

**Table 1:** Overview of individual classifier performance and definition of ensembles

**Classifier**	**Method**	**Features**	**Sensitivity**	**Specificity**	**AUC**	**Ensemble 1**	**Ensemble 2**	**Ensemble 3**	**Ensemble 4**	**Ensemble 5**
Genomics 1	LDA	24	0.73	0.90	0.73	**X**	**X**	**X**		**X**
Genomics 2	SVM	50	0.82	0.95	0.96		**X**			**X**
Genomics 3	RF	50	0.64	0.95	0.92			**X**	**X**	**X**
Genomics 4	EN	43	0.73	1.00	0.93		**X**			**X**
Genomics 5	EN	174	0.73	1.00	0.95				**X**	**X**
Proteomics 1	SVM	12	0.64	0.95	0.94	**X**	**X**	**X**		**X**
Proteomics 2	EN	10	0.64	0.81	0.90			**X**		**X**
Proteomics 3	SVM	33	0.55	0.81	0.83				**X**	**X**
Proteomics 4	EN	13	0.55	0.86	0.85		**X**			**X**
Proteomics 5	SVM	13	0.64	0.95	0.94			**X**		**X**

Shown is a list of 5 genomic and 5 proteomic classifiers, their individual classification performance and their inclusion into 5 ensembles that are explored in this paper. LDA stands for linear discriminant analysis; EN for Elastic Net (Generalized Linear Model); SVM for Support Vector Machine, and RF for Random Forest. Sensitivity, specificity and area under the ROC [receiver operator characteristics] Curve (AUC) for the individual classifiers were estimated using cross-validation.

The development of the Proteomics 1 classifier was described previously [6]. Four additional proteomic classifiers were developed in a process similar to that used for the Genomics analysis described above. Classifiers Proteomics 2–5 in Table 1 are based on EN and SVM classification methods, either robust limma (Proteomics 4–5) or no univariate filter (Proteomics 2–3), and a fold-change cutoff of FC≥1.15 in all cases. In addition, a 75%-rule regarding missing values was implemented, i.e., a protein group was only included if it was detected in at least 75% of all samples. The missing values were imputed using k-nearest neighbours (knn) with *k*=3 across all training samples, independent of class label. Imputation of test samples was performed in each fold of the cross-validation by combining the imputed training data with the test data, then applying knn imputation.

Also shown in Table 1 is the definition of five ensembles representing different combinations of the 10 individual classifiers. Ensemble 1 represents a two-classifier ensemble based on the published genomic and proteomic biomarker panels, Ensemble 2 and 3 expand on Ensemble 1 by adding 2 genomic and 1 proteomic classifier (Ensemble 2), and one genomic and 2 proteomic classifiers (Ensemble 3). Ensemble 4 combines the largest genomic (Genomics 5) and proteomic (Proteomics 3) classifiers and Genomics 3. Ensemble 5 combines all 5 genomic and 5 proteomic classifiers.

The performance of ensemble classifiers was characterized by sensitivity, specificity and AUC. These measures were all derived from a probability-of-AR for the ensemble, which was calculated from probability-of-AR values returned by individual classifiers in the ensemble in combination with either the average probability (AP) or vote-threshold (VT) aggregation methods. For VT a threshold of one was used, i.e., a single AR call by any of the classifiers in the ensemble was enough to call the sample as AR^d^. A probability-threshold of 0.5 was used in the calculation of sensitivity and specificity. Results are summarized in Tables 2 and 3.

**Table 2:** Summary of classification performance for the Average Probability aggregation method

**AVERAGE PROBABILITY**	**Sensitivity**				**Specificity**				**AUC**			
	**Ensemble classifier**	**Individual classifiers**			**Ensemble classifier**	**Individual classifiers**			**Ensemble classifier**	**Individual classifiers**		
		**min**	**max**	**average**		**min**	**max**	**average**		**min**	**max**	**average**
**Ensemble 1**	0.73	0.64	0.73	0.68	0.90	0.90	0.95	0.93	0.95	0.73	0.94	0.84
**Ensemble 2**	0.82	0.55	0.82	0.69	0.95	0.86	1.00	0.93	0.98	0.73	0.96	0.88
**Ensemble 3**	0.73	0.64	0.73	0.65	0.95	0.81	0.95	0.91	0.97	0.73	0.94	0.88
**Ensemble 4**	0.82	0.55	0.73	0.64	0.90	0.81	1.00	0.92	0.97	0.83	0.95	0.90
**Ensemble 5**	0.82	0.55	0.82	0.66	0.95	0.81	1.00	0.92	0.98	0.73	0.96	0.89

Shown is classification performance as measured by sensitivity, specificity and AUC – for the 5 ensembles defined in Table 1 when using the average probability aggregation method. The minimum, maximum and average performances of individual classifiers in the respective ensemble are included in the table for comparison.

**Table 3:** Summary of classification performance for the Vote Threshold aggregation method

**VOTE THRESHOLD**	**Sensitivity**				**Specificity**				**AUC**			
	**Ensemble classifier**	**Individual classifiers**			**Ensemble classifier**	**Individual classifiers**			**Ensemble classifier**	**Individual classifiers**		
		**min**	**max**	**average**		**min**	**max**	**average**		**min**	**max**	**average**
**Ensemble 1**	0.82	0.64	0.73	0.68	0.86	0.90	0.95	0.93	0.89	0.73	0.94	0.84
**Ensemble 2**	0.91	0.55	0.82	0.69	0.76	0.86	1.00	0.93	0.89	0.73	0.96	0.88
**Ensemble 3**	1.00	0.64	0.73	0.65	0.76	0.81	0.95	0.91	0.90	0.73	0.94	0.88
**Ensemble 4**	0.91	0.55	0.73	0.64	0.81	0.81	1.00	0.92	0.95	0.83	0.95	0.90
**Ensemble 5**	1.00	0.55	0.82	0.66	0.62	0.81	1.00	0.92	0.90	0.73	0.96	0.89

Shown is classification performance for the 5 ensembles defined in Table 1 when using the vote threshold aggregation method. Similarly to Table 2, individual classifier performances are included for comparison.

**Figure 3:**
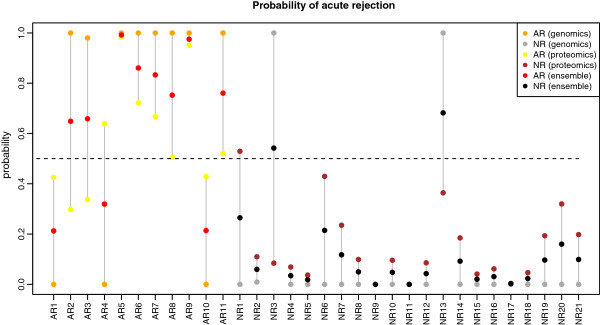
**Comparison of predicted probabilities of acute rejection.** Estimated probability of acute rejection (AR) for each of the AR and NR samples as returned by the Genomics 1 and Proteomics 1 classifiers in Ensemble 1, and the Ensemble 1 classifier which represents a combination of Genomics 1 and Proteomics 1. Samples are grouped along the x-axis into 11 AR (left group) and 21 NR (right group). Each point represents a probability of acute rejection for a specific sample. Three color-coded probabilities are shown per sample. Red and black points represent probabilities from Ensemble 1, orange and grey points from Genomics 1 and yellow and brown points from Proteomics 1.

Ensemble 1 in combination with aggregation method AP achieves a sensitivity and specificity equaling that of the Genomics 1 classifier, while the AUC is improved slightly relative to the Proteomics 1 classifier. Figure 3 shows the estimated probabilities of acute rejection from the different classifiers for each of the 11AR and 21 NR samples. For the 11 AR samples all red and orange pairs fall on the same side of the 0.5-probability threshold line used to determine rejection status. This means that the Genomics 1 and Ensemble 1 classifiers not only display the same sensitivity, but they also misclassify the same AR samples. Also, for the 21 NR samples all black and grey pairs fall on the same side of the 0.5-probability threshold line, which explains the same specificity of Genomics 1 and Ensemble 1, again due to the same NR samples being misclassified. The figure also provides an explanation for the improved AUC of the ensemble as compared to that of Genomics 1 alone. It is due to the probability of the misclassified NR samples being reduced from 1.0 (grey points) to a smaller value (black points), in one case close to the 0.5-probability line. In other words, although the same two NR samples remain misclassified, the AUC of the ensemble is improved because AUC is calculated based on the order of probability-of-AR for all samples. Overall, ensemble 1 in combination with aggregation method AP does not seem to improve classification performance much beyond that of the Genomics 1 classifier alone.

Figure 3 can also be used to interpret the results for Ensemble 1 when the VT aggregation method is used. In this case the red and black points in Figure 3 should be ignored and the ensemble-produced probability of AR is instead given by the larger of the probability-pairs represented by the orange and yellow (for AR), or grey and brown points (for NR). Ensemble 1 has better sensitivity than either the genomic or proteomic classifier alone, and misclassifies only 2 AR samples. However, this improvement comes at the cost of decreased specificity, with 3 misclassified NR samples, as compared to the genomic (2 misclassified) or proteomic (1 misclassified) classifier alone.

For all 10 analyses (5 ensembles with 2 aggregation methods each), we find that sensitivity always meets or exceeds the maximum sensitivity of all individual classifiers in the corresponding ensemble, but exceeds the maximum value for all ensembles wherein the vote-threshold aggregation method is used. A similar observation holds for Ensemble 4 when the AP aggregation method is used and an increased sensitivity of 82% is observed. Specificity, on the other hand, is never better than the best specificity of all individual classifiers in an ensemble, but is always within the min/max range for the 5 ensembles when the AP aggregation method is used, or is usually below the min/max range when the VT aggregation method is used. Ensemble 4 is again the exception here, achieving specificity equaling the minimum value of 81%.

When measuring classifier performance, it can be informative to look at performance in a threshold-independent manner. The area under the curve (AUC) in the ROC assesses performance in this way, summarizing a classifier’s ability to separate two classes across the complete range of possible probability-thresholds. Using this measure of performance, we find that the AUC of ensembles based on the AP aggregation method is always higher than the best (maximum) AUC of the individual classifiers in the corresponding ensemble, although the improvement is generally small as can be seen in Table 2. The AUC when using the VT aggregation method is typically within the range for individual classifiers, but for Ensemble 4 with an AUC of 0.952 slightly exceeds the best individual AUC of 0.948.

Comparing ensemble performance with mean performance of individual classifiers in Tables 2 and 3 shows that the sensitivity and AUC is always higher in the ensemble classifiers, while ensemble specificity is below mean specificity for all 5 ensembles with VT aggregation, and 2 out of 5 ensembles with AP aggregation.

**Figure 4:**
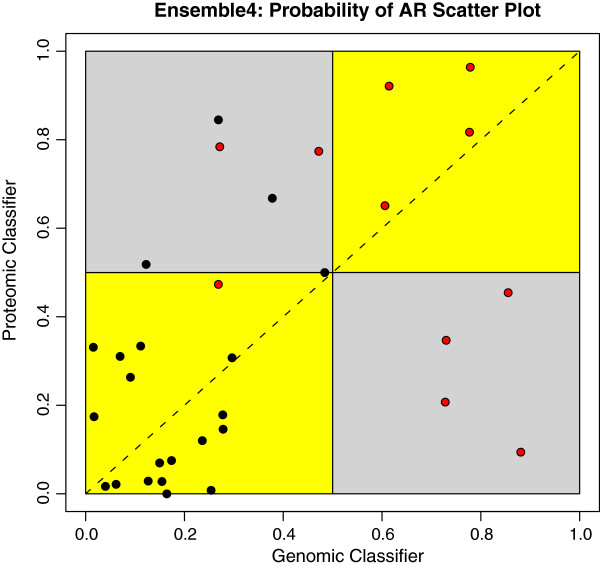
**Classifier comparison within Ensemble 4.** Scatter plot of the predicted posterior probabilities of AR from the Genomics 5 and Proteomics 3 classifier in Ensemble 4. Red points represent 11 AR samples, while black points represent 21 NR samples. Points that fall into yellow areas were classified identically to the genomic and the proteomic classifiers while points in the grey area were classified differently. AR samples are classified correctly when the probability for the corresponding red point is at or above 0.5. NR samples are predicted correctly when the probability is below 0.5.

**Figure 5:**
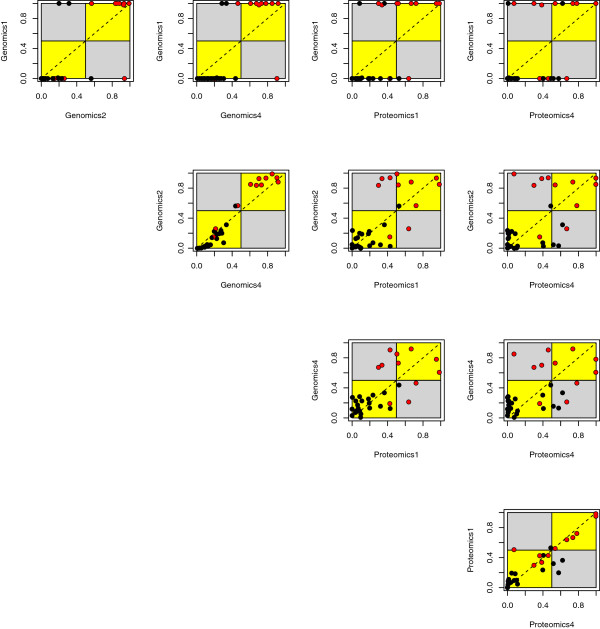
**Comparison of all classifier pairs in Ensemble 2.** Shown is a matrix of scatter plots of the predicted probabilities of AR for all 10 pairs of classifiers in Ensemble 2 as defined in Table 1. Red and black points indicate AR and NR samples respectively, the interpretation of yellow and grey areas is the same as Figure 4.

In Figure 4, one of the two genomic classifiers (Genomics 5) in Ensemble 4 is compared with the proteomic classifier from the same ensemble, using posterior probabilities of acute rejection (AR). The plot demonstrates that for the majority of samples the two classifiers agree and assign the same class label (points that fall in yellow areas), although they do not produce the same probabilities (which would place points on the diagonal line); in some cases, the classifiers disagree on the class of a particular sample (points that fall in grey areas). For example, the proteomic classifier misclassifies the 4 AR samples in the right bottom quadrant, while the genomic classifier misclassifies the two AR samples in the top left quadrant. One AR sample in the bottom left (yellow) square is misclassified by both classifiers. It is possible to compare all pairs of classifiers in an ensemble using the scatter plot approach from Figure 4. An example of this is shown in Figure 5, which displays a matrix of scatter plots for all 10 possible pairs of individual classifiers from Ensemble 2.

In addition to the presented case study, the ensemble framework was also applied to a set of publicly available mRNA- and miRNA- data that contain samples from a variety of human cancers as well as samples for comparable normal tissue. We focused on six tissue types (colon, kidney, prostate, uterus, lung and breast) and used all tumour and normal samples for which both mRNA and miRNA data were available. This resulted in 57 samples (38 tumour and 19 normal samples). The computational pipeline was applied, using 10x 19-fold cross-validation and a maximum AUC (within one standard-error) model selection criteria to develop a set of 12 classifiers for each of the mRNA- and miRNA- data sets separately. Classifier characteristics and estimated performances are shown in Table 4, together with the definition of six ensembles that represent different combinations of mRNA- and miRNA- classifiers. Similar to our results, previous work by Ramaswamy et al. on a super-set of the mRNA-data was able to differentiate tumour- from normal samples with an accuracy of 92% using SVM and cross-validation [41].

**Table 4:** Overview of individual classifier performance and definition of ensembles

**Classifier**	**Method**	**Features**	**Accuracy**	**Sensitivity**	**Specificity**	**AUC**	**Ensemble A**	**Ensemble B**	**Ensemble C**	**Ensemble D**	**Ensemble E**	**Ensemble F**
mRNA-Classifier1	EN	182	0.9298	0.9737	0.8421	0.9737	X			X	X	
mRNA-Classifier2	EN	73	0.9123	1.0000	0.7368	0.9709					X	
mRNA-Classifier3	EN	36	0.8947	0.9737	0.7368	0.9501			X		X	
mRNA-Classifier4	LDA	2	0.9298	0.9211	0.9474	0.9640					X	
mRNA-Classifier5	RF	500	0.8947	0.9737	0.7368	0.9418					X	
mRNA-Classifier6	SVM	500	0.9298	0.9474	0.8947	0.9640					X	
mRNA-Classifier7	EN	43	0.9123	0.9474	0.8421	0.9598						X
mRNA-Classifier8	EN	25	0.9298	0.9737	0.8421	0.9612						X
mRNA-Classifier9	EN	17	0.9298	0.9737	0.8421	0.9695						X
mRNA-Classifier10	LDA	2	0.9298	0.9211	0.9474	0.9640				X		X
mRNA-Classifier11	RF	50	0.9298	0.9474	0.8947	0.9584			X			X
mRNA-Classifier12	SVM	50	0.8947	0.9211	0.8421	0.9557		X		X		X
miRNA-Classifier1	EN	66	0.8947	0.9211	0.8421	0.9626	X				X	
miRNA-Classifier2	EN	21	0.9474	0.9737	0.8947	0.9709			X		X	
miRNA-Classifier3	EN	8	0.9649	0.9737	0.9474	0.9723				X	X	
miRNA-Classifier4	LDA	4	0.9298	0.9211	0.9474	0.9626				X	X	
miRNA-Classifier5	RF	152	0.8947	0.8947	0.8947	0.9765					X	
miRNA-Classifier6	SVM	152	0.9123	0.9474	0.8421	0.9626					X	
miRNA-Classifier7	EN	36	0.9298	0.9474	0.8947	0.9709						X
miRNA-Classifier8	EN	16	0.9298	0.9474	0.8947	0.9848						X
miRNA-Classifier9	EN	12	0.9474	0.9737	0.8947	0.9806						X
miRNA-Classifier10	LDA	4	0.9298	0.9211	0.9474	0.9626						X
miRNA-Classifier11	RF	50	0.9123	0.9211	0.8947	0.9778				X		X
miRNA-Classifier12	SVM	50	0.8947	0.9211	0.8421	0.9612		X	X			X

Shown is a list of 12 mRNA- and 12 miRNA classifiers, their individual classification performance and their inclusion into 6 ensembles that are explored for classification of tumour vs normal samples. Abbreviations are the same as in Table 1.

**Figure 6:**
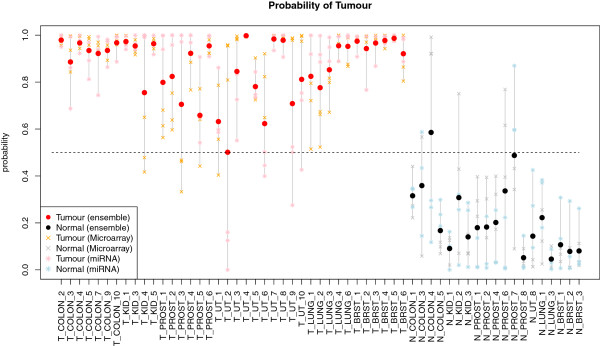
**Comparison of predicted probabilities of tumour.** Estimated probability of tumour for each of the tumour- and normal samples as returned by all six classifiers in Ensemble D, and the Ensemble D classifier itself. Samples are grouped along the x-axis into 38 tumour (left) and 19 normal (right). Seven color-coded probabilities are shown per sample. Red and black points represent probabilities from Ensemble D, orange and grey crosses from the three mRNA classifiers, and pink and blue stars from the three miRNA classifiers.

Performance of ensemble classifiers was then determined for the AP and VT aggregation methods. The vote threshold was set to one as before, i.e. a sample was classified to class *tumour* if at least one of the classifiers in the ensemble classified it as such. Classification performance is summarized in Table 5 (AP) and Table 6 (VT). For both AP and VT aggregation methods, all ensembles achieve a higher AUC than the best individual classifier in the respective ensemble. Ensembles D and F with the AP aggregation method show the best performances, both having sensitivity of 100%, specificity of 95% and AUC of 0.9986, although ensemble F is based on twice as many individual classifiers as ensemble D. For both ensembles, only one normal sample is misclassified as can be seen in Figure 6, which shows the probability of tumour for ensemble D and for the six individual classifiers that are equally split between mRNA- and miRNA-classifiers (three each).

**Table 5:** Summary of classification performance for the Average Probability aggregation method

**AVERAGE PROBABILITY**	**Sensitivity**				**Specificity**				**AUC**			
	**Ensemble classifier**	**Individual classifiers**			**Ensemble classifier**	**Individual classifiers**			**Ensemble classifier**	**Individual classifiers**		
		**min**	**max**	**average**		**min**	**max**	**average**		**min**	**max**	**average**
**Ensemble A**	1.0000	0.9211	0.9737	0.9474	0.8421	0.8421	0.8421	0.8421	0.9972	0.9626	0.9737	0.9681
**Ensemble B**	0.9737	0.9211	0.9211	0.9211	0.8421	0.8421	0.8421	0.8421	0.9931	0.9557	0.9612	0.9584
**Ensemble C**	1.0000	0.9211	0.9737	0.9539	0.8421	0.7368	0.8947	0.8421	0.9917	0.9501	0.9709	0.9602
**Ensemble D**	1.0000	0.9211	0.9737	0.9386	0.9474	0.8421	0.9474	0.9035	0.9986	0.9557	0.9778	0.9677
**Ensemble E**	1.0000	0.8947	1.0000	0.9518	0.8947	0.7368	0.9474	0.8553	0.9972	0.9418	0.9765	0.9643
**Ensemble F**	1.0000	0.9211	0.9737	0.9430	0.9474	0.8421	0.9474	0.8816	0.9986	0.9557	0.9848	0.9672

Shown is performance for tumour vs normal classification for the 6 ensembles defined in Table 4 using the average probability aggregation method. The minimum, maximum and average performances of individual classifiers in the respective ensemble are included in the table for comparison.

**Table 6:** Summary of classification performance for the Vote Threshold aggregation method

**VOTE THRESHOLD**	**Sensitivity**				**Specificity**				**AUC**			
	**Ensemble classifier**	**Individual classifiers**			**Ensemble classifier**	**Individual classifiers**			**Ensemble classifier**	**Individual classifiers**		
		**min**	**max**	**average**		**min**	**max**	**average**		**min**	**max**	**average**
**Ensemble A**	1.0000	0.9211	0.9737	0.9474	0.7368	0.8421	0.8421	0.8421	0.9875	0.9626	0.9737	0.9681
**Ensemble B**	1.0000	0.9211	0.9211	0.9211	0.6842	0.8421	0.8421	0.8421	0.9917	0.9557	0.9612	0.9584
**Ensemble C**	1.0000	0.9211	0.9737	0.9539	0.6842	0.7368	0.8947	0.8421	0.9861	0.9501	0.9709	0.9602
**Ensemble D**	1.0000	0.9211	0.9737	0.9386	0.7368	0.8421	0.9474	0.9035	0.9875	0.9557	0.9778	0.9677
**Ensemble E**	1.0000	0.8947	1.0000	0.9518	0.6316	0.7368	0.9474	0.8553	0.9903	0.9418	0.9765	0.9643
**Ensemble F**	1.0000	0.9211	0.9737	0.9430	0.6842	0.8421	0.9474	0.8816	0.9931	0.9557	0.9848	0.9672

Shown is performance for tumour vs normal classification for the 6 ensembles defined in Table 4 using the vote threshold aggregation method. Similarly to Table 5, individual classifier performances are included for comparison.

From Figure 6, it can be seen across the 57 samples that the three classifiers based on mRNA-data show similar probabilities of tumour most of the time, as do the three classifiers based on miRNA-data. However, because miRNA-classifiers perform better when mRNA-classifiers misclassify (for example in several of the prostate cancer samples), and mRNA-classifiers perform better when some of the miRNA classifiers misclassify (for example in several of the uterus cancer samples), the ensemble can overall benefit from the averaging of probabilities. This is evident by the fact that all ensemble probabilities for the cancer samples (red points) fall above the probability=0.5 dashed line, thus achieving the aforementioned sensitivity of 100%. A similar effect of probability-grouping by platform is observed for the normal samples. For example, the mRNA-classifiers show a probability of tumour>0.9 for the single misclassified normal sample, while all miRNA-classifiers have a probability of less than 0.3 for the same sample.

## Discussion

A biomarker development pipeline with applications in genomic-, proteomic-, and other –omic data was presented and applied to the clinical challenge of classifying acute renal allograft rejection in blood samples. Genomic- and proteomic-based classification models were developed and showed adequate classification performance for clinical use. Individual genomic- and proteomic-based classifiers were then combined into Ensemble classifiers. Given the cited improvement in classification performance of ensemble classifiers in other fields [36,42-44], an important question underlying our analysis was the extent that ensembles can improve classification performance regarding acute renal allograft rejection beyond that of individual genomic and proteomic classifiers alone. Our application area is characterized by small sample sizes and adequate classification performance of individual classifiers. In general, we found that classification performance improved by using ensembles, although improvements in some performance measures might be countered by a decrease in other performance measures. In general, the number of classifiers in an ensemble did not seem to affect performance improvements.

When diagnosing acute kidney rejection, it is arguably more important to avoid false negatives (rejection that is falsely classified as non-rejection) than false positives (non-rejection falsely classified as rejection), because delays in the treatment of acute rejection cause both short- and long-term harm to the patient [45,46]. This was the motivation behind the vote-threshold aggregation method, which ensures that a single individual classifier vote for acute-rejection would result in an acute-rejection classification by the ensemble. The results in Table 3 demonstrate that the VT aggregation method achieved an increase in sensitivity across all ensembles though at the intuitively expected cost of decreased specificity in 4 out of 5 ensembles. The impact of this approach is similar to lowering the probability-of-AR-threshold for an individual classifier, but it benefits from the increased diversity that comes with an ensemble, which in our case includes genomic- and proteomic-based biological signals. The VT method is especially valuable in cases where one platform is able to detect a rejection signal in some patients while another platform is not, as is demonstrated, for example, in Figure 4.

One of the ensembles (Ensemble 1) represents a two-classifier ensemble combining our previously published genomic and proteomic classifiers [5,6]. Even though AUC improves slightly when using the AP aggregation method, the same samples are misclassified as in the genomic classifier of Ensemble 1. Sensitivity is improved beyond that of the genomic or proteomic classifier alone when the VT aggregation method is used, but specificity dropped below the values for the individual classifiers. Ensemble 1 therefore does not seem to improve classification performance much beyond that of the Genomics 1 classifier alone. Ensembles 2, 3 and 5 represent an extension of Ensemble 1, where further genomic and/or proteomic classifiers were added. For the AP aggregation method these three ensembles show a similar performance range as Ensemble 1, while for the VT aggregation method Ensembles 3 and 5 can improve sensitivity to 100% but drop below the range of individual classifiers for specificity, while staying within range regarding AUC. Ensemble 5 has a specificity of 62% which is the lowest specificity across all 5 ensembles and 10 individual classifiers. This is not surprising since Ensemble 5 combines all 10 individual classifiers and a single AR-classification of one of the 10 classifiers is enough to call the sample AR, therefore maximally increasing sensitivity and lowering specificity. In this case and for ensembles with a larger number of individual classifiers, the VT method might perform better with a higher threshold, which could be, for example, AR-classification from at least two classifiers.

The best-performing ensemble (Ensemble 4) excludes the published genomic and proteomic classifiers but instead combines the largest genomic, the largest proteomic and a 50-feature genomic classifier based on Random Forest. The results in Table 2 and Table 3 favour Ensemble 4, which is the only one that improves sensitivity and AUC beyond that of individual classifiers in the ensemble while staying within the range for specificity. The two genomic classifiers in Ensemble 4 are based on Elastic Net (174 features) and Random Forest (50 features, of which 49 are also included in the 174-Elastic Net classifier). The proteomic classifier is based on SVM using 33 features that were selected by fold-change criteria. A contributing factor for the good performance of Ensemble 4 could therefore be the use of comparatively large classifier panels and a fold-change filter on the proteomic side.

Several parts of the biomarker development pipeline for individual classifiers were designed to reduce the selection of false positive biomarkers, including pre-filtering, multiple hypothesis testing correction, cross-validation to maximize use of the small number of available samples, and nested cross-validation to avoid bias when models are tuned [29,34,47,48]. Ensembles provide an additional layer of robustness for classification when aggregation methods that average over several classifiers, e.g. average probability or majority vote, are used. This robustness is achieved by reducing the impact of inaccurate classifiers based on false positive genes or proteins by allowing more accurate classifiers in the ensemble to “out-vote” a small number of inaccurate classifiers. Related to the previous point is the fact that the kidney rejection data is “wide” data, which is defined as having more features than samples. In “wide” data problems it is not feasible to find the best classifier. Instead, one commonly finds many and possibly quite different classifiers that seem equally valid while displaying a range of classification performances. Ensembling therefore provides a robust approach to “wide” data classification problems.

An important question surrounding ensembling concerns the choice of individual classifiers that should be part of the ensemble. Theoretical analysis points to including classifiers that are as independent as possible [36]. One source of “independence” in the acute kidney rejection case study comes from the two data types, i.e. genomic versus proteomic. Within genomic and proteomic data, classifiers are developed using different combinations of filtering- and classification methods as shown in Figure 1, thus focusing on different aspects of genomic and proteomic data respectively. An additional source of “independence” that has not been explored in this study could be provided on a biological level. Bioinformatics tools, such as pathway analysis tools and ontology-based tools, can provide insights as to how much individual biomarker panels differ biologically. Individual classifiers in an ensemble could then be selected to cover a wide range of biological pathways, thus providing a diverse biological cross-section. Pathway analysis is an area of active research in its own right that is currently going through a dynamic flux [49]. Hence, we have concentrated in our approach and discussion on computational aspects of ensemble classifiers.

In addition to selecting individual classifiers to be combined in an ensemble, a weighting needs to be provided. We have used equal weights of individual classifiers in our analyses, as suggested by the term *average* probability. In general, each classifier can be weighted differently in classifier aggregation such that more trustworthy classifiers receive a higher weight. It is important to note that any composition of an ensemble introduces a form of weighting. For example, an ensemble of 2 genomic and 5 proteomic classifiers, in which all classifiers have equal weights, would put a higher weight on proteomic-based classifiers as a group when compared to genomic-based classifiers. If one prefers to give equal weight to genomic- and proteomic-based classifier-groups, the two genomic-based classifiers should have a weight of 0.25 each (thus adding up to 0.5), while the five proteomic-based classifiers should have a weight of 0.1 each (also adding up to 0.5). The five ensembles in Table 1 followed an underlying balanced design in this regard, i.e., the difference in the number of genomic and proteomic classifiers in an ensemble, is at most 1.

Figure 5 shows a matrix of scatter plots for all 10 possible pairs of individual classifiers from Ensemble 2, demonstrating the usefulness of this type of visualization in providing an overview of the diversity between the classifiers in an ensemble. The scatter plots between pairs of genomic classifiers (three plots in the upper left) and proteomic classifiers (one plot in the bottom right) show similar classification of samples, with most samples falling into the yellow areas. The remaining 6 scatter plots compare one genomic and one proteomic classifier each. Here, an increase in disagreement between the classifier pairs is observed, which is evident by more samples falling in the grey areas. Disagreement to some extent is desired in ensemble classifiers since they derive a benefit from the diversity of the underlying classifiers. In addition to comparing classifiers in an ensemble based on the number of features and individual performance characteristics as shown in Table 1, one can also use information from scatter plots as shown in Figure 4 and 5 to add or remove classifiers, in an effort to optimize diversity during the ensemble design process. It should be noted that the number of plots in a scatter plot matrix grows with the square of the number of individual classifiers, an effect that poses a practical limitation on this type of visualization.

Because the proteo-genomic ensemble approach assumes fully developed individual classifiers, test samples need to be classified by genomic and proteomic classifiers before they can be aggregated. This requires the samples to be run on both platforms. In cases where a sample is only run on one platform, the ensemble classifier cannot be used. An alternative in this case is to fall back on a platform-specific classifier, which by itself could be an ensemble (e.g., a genomic-ensemble), although one would lose the advantage of using information from diverse sources for classification. The inclusion of data from other platforms within the presented ensemble framework, for example miRNA, metabolomic or clinical data sources, is easily possible as long as patient-matched measurements from the corresponding platforms are provided. The generality of the ensemble framework has been demonstrated by applying it off the shelf to an additional cancer data set based on two different types of genomic data (mRNA and miRNA). The findings there show that ensemble classifiers can improve upon already well-performing individual mRNA and miRNA classifiers, thus supporting the notion that ensemble classifiers based on a diverse set of individual classifiers across different platforms have the ability to outperform any single classifier in the ensemble.

## Conclusions

Proteo-genomic biomarker ensemble classifiers show promise in the diagnosis of acute renal allograft rejection and can improve classification performance beyond that of individual genomic or proteomic classifiers alone. The Vote Threshold application method allows fine-tuning of sensitivity and specificity while incorporating diverse classification signals from individual classifiers. This is an important feature in application areas where sensitivity is more important than specificity. Validation of our renal allograft rejection results in an international multicenter study is currently underway.

## Endnotes

^a^The Genomics 1 classifier was developed based on 33-samples which included one additional non-rejection sample that was available only on the genomic platform. This sample was not included in the development of the other genomic and proteomic classifiers.

^b^Classifier Proteomics 1 in Table 1 is from a previous publication [6] which used a 67% minimum detection rule.

^c^Performance estimates for classifier Genomics 1 in Table 1 were based on values for 32 samples derived from 11-fold cross-validation of the 33 sample set as described in a previous publication [5].

^d^Performance estimates for ensembles that included the Genomics 1 classifier used posterior probabilities for the 32 samples in common.

## Abbreviations

AR: Acute Rejection; NR: Non-Rejection; LDA: Linear Discriminant Analysis; SVM: Support Vector Machine; EN: Elastic Net (Generalized Linear Model); RF: Random Forest; ROC: Receiver Operating Characteristics; AUC: Area under the (ROC) Curve; PGCA: Protein Group Code Algorithm; AP: Average Probability; VT: Vote Threshold; mRNA: Messenger RNA; miRNA: MicroRNA.

## Competing interests

The authors declare that they have no competing interests.

## Authors’ contributions

OG carried out computational genomic- and ensemble analyses, participated in the design, execution and analytical discussions of the work, and prepared the manuscript. VC carried out the computational proteomic analysis and contributed to the design, execution and analytical discussions of the work presented in this manuscript. GCF contributed to the design, development and description of the proteomics pipeline, analytical discussion of the work and reviewing the manuscript. RB contributed to conception, design and statistical discussion of the computational pipeline and ensembles discussed in the manuscript. ST contributed to the design and participated in analytical and biologically discussions of the work. ZH contributed to design and analytical discussion of the computational pipeline, and data management support. MT participated in analytical discussion of the work and preparation of iTRAQ proteomics data. RM participated in the conception and design of the work discussed in this manuscript. BM contributed to the conception, design, execution and analytical discussions of the work discussed in this manuscript. PK participated in the conception, design and execution of the work discussed in this manuscript. RN contributed to the conception, design, execution and analytical discussions of the work, and participated in preparing the manuscript. All authors read and approved the final manuscript.
